# Kindlin Regulates Mechanosensitive Activation and Adhesion Assembly of Integrin beta6

**DOI:** 10.1002/advs.202501078

**Published:** 2025-06-10

**Authors:** Wan Ning Lee, Jiamin Li, Nan‐Peng Chen, Cheng‐han Yu

**Affiliations:** ^1^ School of Biomedical Sciences Li Ka Shing Faculty of Medicine The University of Hong Kong Pokfulam Hong Kong SAR China; ^2^ Shenzhen Medical Academy of Research and Translation (SMART) Shenzhen Guangdong 518132 China; ^3^ Institute of Systems and Physical Biology Shenzhen Bay Laboratory Shenzhen Guangdong 518132 China; ^4^ Westlake University Hangzhou China

**Keywords:** integrin activation, mechanosensitive cell adhesion, kindlin

## Abstract

Inside‐out and outside‐in activations of integrin both play important roles in adhesion formation and cell motility. While such mechanisms of RGD‐binding integrin, including integrin β1 and β3, are well documented, mechanosensitive regulations of integrin β6 remain largely unknown. Using traction force‐free RGD‐membrane as the model system, integrin β6 and viscous RGD ligands initially colocalize, assemble micrometer‐sized clusters (15‐min), and then gradually dissipate (60‐min). Domain‐swapping approaches are used and find that integrin β6 chimera with integrin β1's cytoplasmic tail can establish the persistent spatial clustering, suggesting the involvement of inside‐out signaling. In vitro, pulldown and microscale thermophoresis experiments reveal that cytoplasmic adapter kindlin2 is the key factor and specifically exhibits weak association with integrin β6. Such kindlin2 deficiency can in turn be restored by additional kindlin2 or the introduction of PIPK1 that promotes kindlin2 recruitment to the plasma membrane. Dissipated integrin β6 is found in non‐active conformation. Strengthening the interaction between kindlin2 and integrin β6 stabilizes integrin β6 in active conformation and is necessary to promote the migration of AsPC‐1 cells on the soft PDMS. Thus, kindlin‐mediated inside‐out activation of integrin β6 bypasses extracellular force‐dependent signaling and regulates the mechanosensitive adhesion assembly and cell migration on the compliant substrate.

## Graphical abstract

Extracellular matrix compliance plays a critical role in the regulation of integrin‐mediated adhesion and cell motility. Here, it is reported that strengthening the kindlin2‐integrin β6 interaction overcomes the dependence on extracellular substrate stiffness and promotes integrin β6‐mediated cell migration. In general, kindlin‐mediated inside‐out activation can functionally modulate the dynamic reorganization of integrin receptors and provide new insights into the regulation of mechanosensitive adhesion signaling.

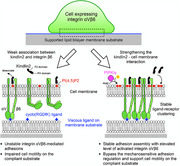

## 1 Introduction

Cell‐matrix adhesions are vital for tissue integrity and regeneration. Spanning across the plasma membrane, integrin receptors are the key components of cell‐matrix adhesion and convert extracellular biochemical and biomechanical stimuli into intracellular signal cascades, including actin cytoskeleton remodeling, Src/FAK kinase activation, and phosphoinositide biogenesis.^[^ ^1^, ^2^ ^]^ Each integrin receptor is a heterodimer and is composed of an alpha (α) and a beta (β) subunit. There are eighteen α and eight β subunits in mammalian cells, and 24 types of integrin heterodimer combinations have been reported. In particular, integrin αV subunit can dimerize with β1, β3, β5, β6, or β8 subunit and form a specific heterodimer subgroup that binds to the arginine‐glycine‐aspartic acid (RGD) motif found in fibronectin and other extracellular matrix proteins.^[^ ^3^ ^]^ Integrin heterodimers adopt structurally distinct conformations with different ligand binding affinity. In the ligand‐unbound state, inactive integrin heterodimers mainly stay in the bent conformation extracellularly and have a limited exposure of cytoplasmic domain.^[^ ^4^, ^5^ ^]^ Integrin activation, involving the shift from bent‐closed (low affinity) to extended‐open (high affinity) state, can be induced upon extracellular ligand binding.^[^ ^6^ ^]^ In the extended‐open conformation, the cytoplasmic domain of integrin β subunit becomes accessible to the key adapter proteins, such as kindlin and talin. Additional signaling adapter proteins are further recruited to activate integrins, contributing to the formation of adhesion sites at the cell‐matrix interface.^[^ ^7^ ^]^

Extracellular ligand binding (outside‐in) and intracellular adapter protein association (inside‐out) are both essential for integrin activation and adhesion assembly.^[^ ^8^, ^9^ ^]^ Kindlin and talin are essential adapter proteins of integrin, as either kindlin or talin knockout cells fail to attach to the matrix‐coated substrate.^[^ ^10^ ^]^ The N‐terminal head domain of talin binds to the membrane‐proximal NPxY motif in the cytoplasmic tail of integrin β subunit, playing a crucial role in promoting integrin activation. Meanwhile, the C‐terminal rod domain of talin features multiple F‐actin binding sites.^[^ ^11^, ^12^ ^]^ When the contractile force is applied, talin can be subjected to the unfolding process, and the crypted vinculin binding sites in the rod domain of talin become exposed.^[^ ^13^ ^]^ As a result, vinculin is recruited to the force‐bearing adhesion sites and further stabilizes the association of talin to the cytoplasmic tail of integrin.^[^ ^14^, ^15^ ^]^ In mammalian tissues, there are three kindlin isoforms with non‐redundant functions. Kindlin1 is mainly found in the epithelium, whereas kindlin2 is widely expressed but absent in the hematopoietic tissue.^[^ ^16^ ^]^ Kindlin3 expression is limited to hematopoietic and endothelial cells.^[^ ^17^ ^]^ In terms of domain homology, all kindlin isoforms start with an F0 domain, followed by F1, F2, and F3 domains that are homologous to the domains found in 4.1‐ezrin‐radixin‐moesin (FERM) protein family. While kindlin lacks direct interactions with cytoskeleton, F3 domain specifically binds to the membrane‐distal NxxY motif in the cytoplasmic tail of integrin β subunit and supports the active conformation of integrin.^[^ ^18^, ^19^ ^]^ Recent NMR‐based study has revealed that the direct interactions between kindlin2's F0 domain and talin1's head domain play a critical role in overcoming low talin affinity for the kindlin2‐occupied cytoplasmic tail of integrin β subunit. In addition, kindlin binding to the extended‐open conformation of integrin can consequently lead to high talin affinity for the membrane‐proximal NPxY motif.^[^ ^20^, ^21^ ^]^ Despite its importance in integrin activation, the functional role of kindlin in regulating mechanosensitive adhesion assembly remains unclear.

While activated by the same extracellular ligand, various integrins can lead to divergent intracellular adhesion signals. On the rigid fibronectin‐coated substrate, integrin β1 induces traction force generation, whereas integrin β3 mediates the formation of large focal adhesions.^[^ ^22^, ^23^ ^]^ Apart from biochemical signals, biomechanical cues in the extracellular matrix also play an important role in modulating the spatiotemporal assembly of integrin‐mediated adhesions.^[^ ^24^, ^25^ ^]^ Integrin β6 binds to the same RGD‐containing extracellular ligands, but the mechanosensitive characteristics of integrin β6 adhesion have not been explored. Supported lipid bilayer membrane is a liquid‐like viscous surface, and the ligand functionalized over the membrane on the cover glass can exhibit 2D fluidity over a sub‐centimeter distance.^[^ ^26^ ^]^ As a model system of traction force‐free substrate, the viscous RGD‐membrane with defined ligand density has been widely used to decouple various mechanosensitive signal transductions at the integrin‐mediated adhesion.^[^ ^27^, ^28^ ^]^ In this study, we utilize viscous RGD‐membrane and stiffness‐defined substrates to unveil the kindlin2‐mediated mechanosensitive assembly of integrin β6 adhesion and its implication in cell migration.

## 2 Result

### 2.1 Integrin β6 Adhesions on Viscous RGD‐Membrane Gradually Dissipate

**Figure 1:**
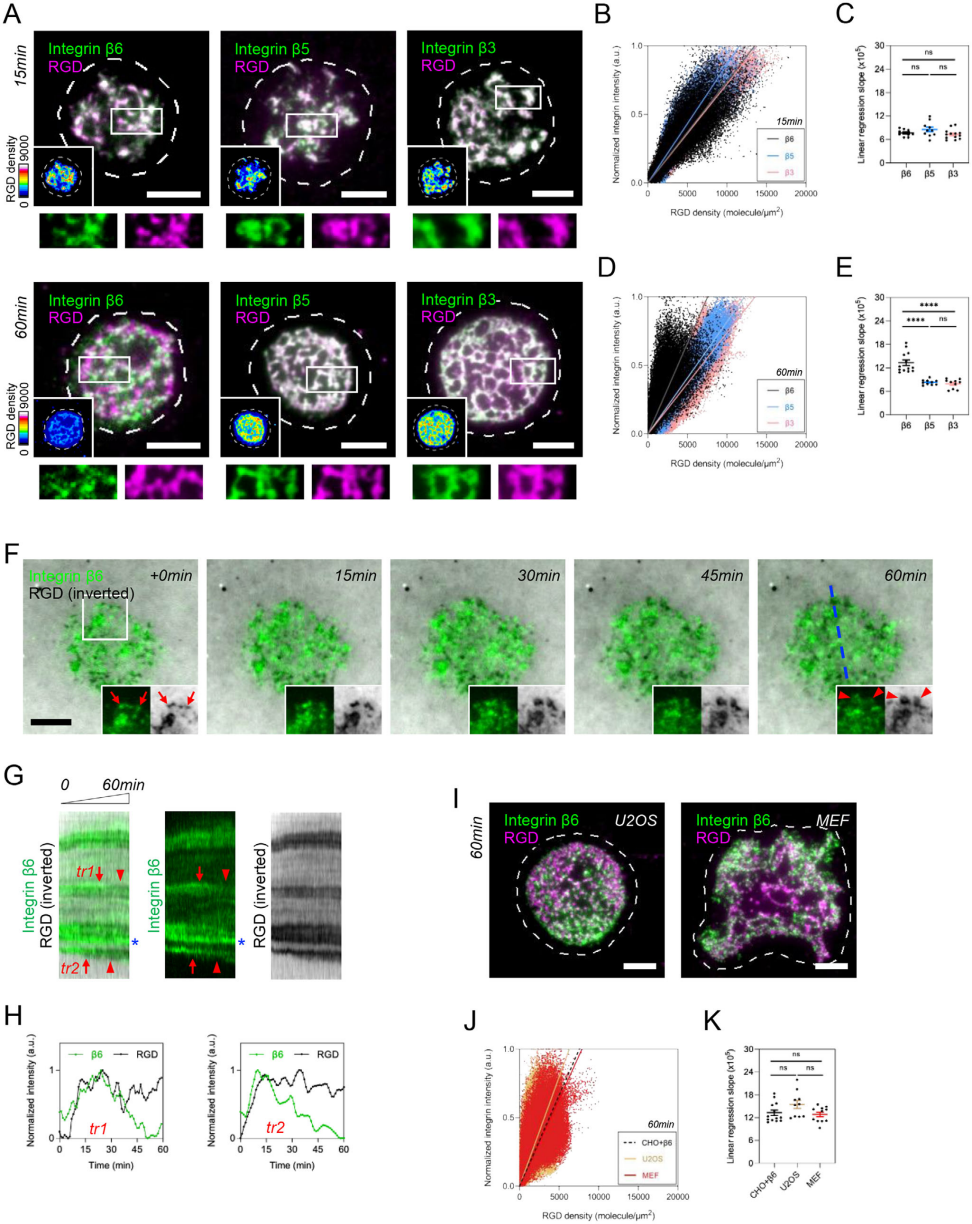
Initial integrin β6 clusters gradually dissipate on viscous RGD‐membrane. A) EGFP‐tagged integrin β6, β5, and β3 expressed in CHO‐B2 cells nucleate RGD clusters in 15 and 60‐min. Pseudocolor panels indicate the corresponding densities of RGD ligand. The inset shows the boxed region (5 × 2 µM^2^). B) The scatter plot of RGD density and integrin intensity in 15‐min. C) The slopes of linear regression in B (cell number N; β6, N=10; β5, N=10; β3, N=13). D) The scatter plot of RGD density and integrin intensity in 60‐min. E) The slopes of linear regression in D (cell number N; β6, N=13; β5, N=10; β3, N=10). The increasing slope indicates the dissociation of integrin β6 in 60‐min, compared to integrin β5 and β3. F) EGFP‐tagged integrin β6 in live CHO‐B2 cells initially assembles micron‐sized adhesions (arrow) and subsequently dissociates from RGD clusters (arrowhead; 60‐min). RGD intensity is shown inverted. The inset shows the boxed region (5.5 × 5.5 µM^2^). G) The kymograph along the dashed line in F. Initial integrin β6‐GFP adhesions (arrow) gradually dissipate (arrowhead). The loss of integrin β6 does not result from photobleaching (asterisk). H) The intensity profiles of integrin β6 and RGD along tr1 and tr2. I) EGFP‐tagged integrin β6 in U2OS and MEF cells also poorly colocalizes with RGD clusters in 60‐min. J) The scatter plot of RGD density and integrin intensity in U2OS and MEF cells in 60‐min. The dashed line denotes the linear regression of integrin β6 (CHO+β6; See Figure 1D). K) The slopes of linear regression in J (cell number N; U2OS, N=10; MEF, N=11). All experiments have been independently repeated three times. Error estimates are S.E.M. The statistical information is in Table S4. One‐way analysis of variance (ANOVA) is used for the statistical analysis. not significant, ns; *P* > 0.1234; and ^****^ *P* < 0.0001. Scale bars represent 5 µM.

CHO‐B2 cells natively express integrin αV but lack the expression of integrin β6 (Figure S1A, Supporting Information). When plated on the viscous RGD‐membrane, CHO‐B2 cells expressing EGFP‐tagged integrin β6, β5, and β3 promptly adhered and formed dense clusters with RGD ligands in 15‐min (**Figure** **1**A–C). Integrin β5 and β3 were consistently enriched at the adhesions throughout 60‐min. Integrin β6, by contrast, became dissociated from RGD clusters (Figure 1A). To quantify the dissociation of integrin β6, we first measured the intensity‐density relationship of RGD by titration and quantitative fluorescence microscopy (Figure S1B, Supporting Information). We then employed the pixel‐wise intensity analysis to quantitatively compare the intensity correlation between RGD and integrin (see Experimental Section). After background subtraction, the intensity values of each pixel were then used to construct the scatter plot of RGD density and integrin. Overall, the densities of RGD ligand nucleated by all integrins in the early phase of cell adhesion (15‐min) were higher than those of late adhesions (60‐min). However, integrin β6, rather than integrin β5 and β3, exhibited a distinct increase of linear regression slope in 60‐min, indicating the dissociation of integrin β6 from RGD clusters (Figure 1B–E). Such integrin β6 adhesion discrepancy did not result from the overexpression artifact nor ligand‐receptor endocytosis (Figure S1C–H, Supporting Information). Through live‐cell imaging, integrin β6‐GFP in live CHO‐B2 cells gradually dissipated from the pre‐existing RGD clusters and exhibited a dispersed pattern (Figure 1F–H). Similar dissociations between integrin β6 and RGD ligands were also observed in other cell types, such as U2OS and MEF cells (Figure 1I–K).

### 2.2 Cytoplasmic Domain of Integrin β6 Determines the Spatiotemporal Adhesion Assembly

**Figure 2:**
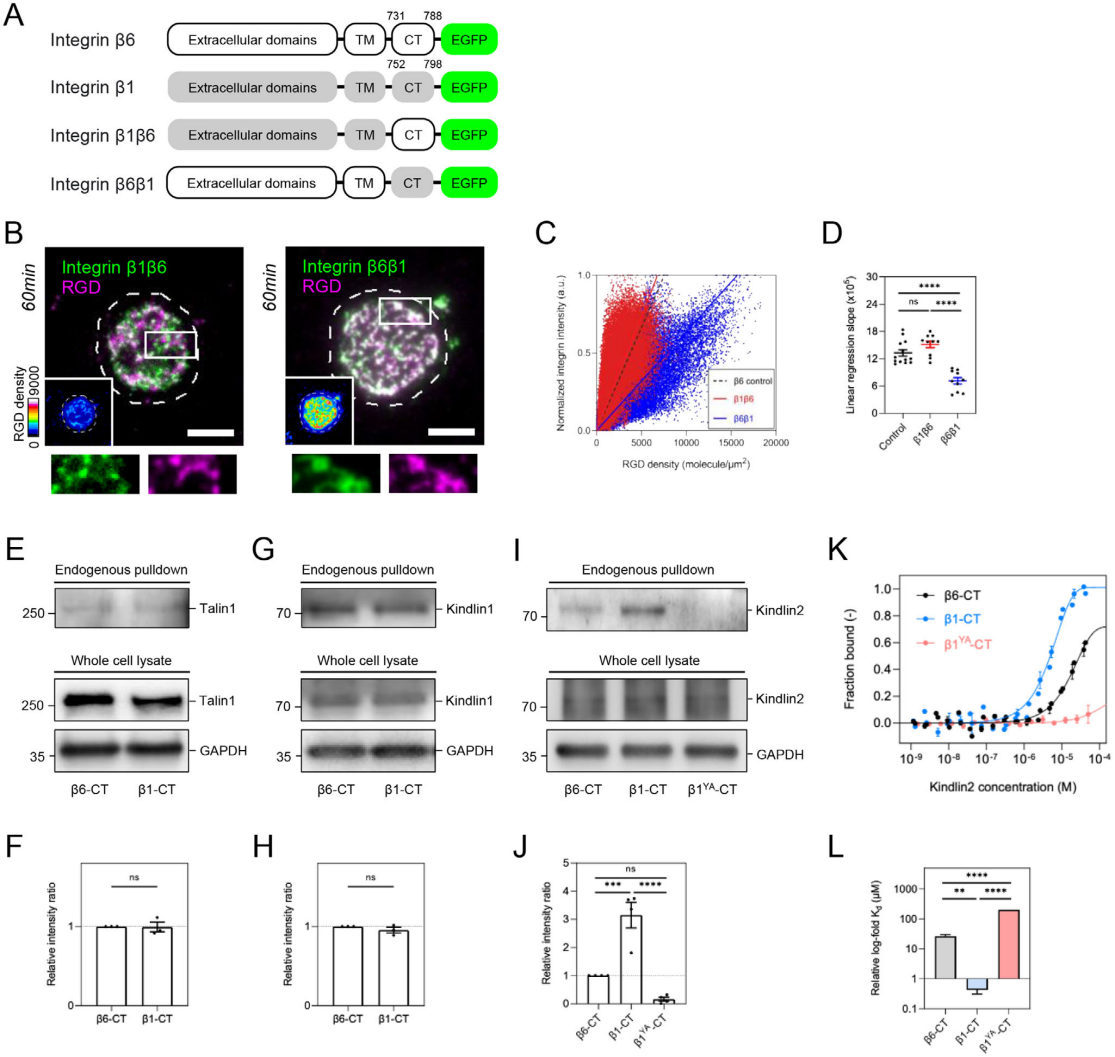
Cytoplasmic domain determines the stability of integrin β6 adhesion. A) Schematic diagrams of integrin β6, β1, and domain swapping chimeras. B) Integrin β6β1‐GFP, rather than integrin β1β6‐GFP, remains stably associated with RGD clusters in 60‐min. Pseudocolor panels indicate the corresponding densities of RGD ligand. The inset shows the boxed region (5 × 2 µM^2^). C) The scatter plot of RGD density and integrin intensity as indicated. The dashed line denotes the linear regression of integrin β6 control (see Figure 1D). D) The slopes of linear regression in C (cell number N; β1β6, N=10; β6β1, N=10). E,F) Western blots and quantification of talin1 pulled down by the cytoplasmic tail of integrin β6 and integrin β1. G,H) Western blots and quantification of kindlin1 pulled down by the cytoplasmic tail of integrin β6 and integrin β1. I,J) Western blots and quantification of kindlin2 pulled down by the cytoplasmic tail of integrin β6, integrin β1, and integrin β1^Y795A^. Integrin β6 exhibits a weaker association with kindlin2, compared to integrin β1. K,L) MST measurements of kindlin2 affinity with the cytoplasmic tail of integrin β6, integrin β1, or integrin β1^Y795A^. All experiments have been independently repeated three times. Error estimates are S.E.M. The statistical information is in Table S4 (Supporting Information). Unpaired two‐tailed Student's *t*‐test and one‐way analysis of variance (ANOVA) are used for the statistical analysis. not significant, ns; *P* > 0.1234; ^**^ *P* < 0.0021; ^***^ *P* < 0.0002; and ^****^ *P* < 0.0001. Scale bars represent 5 µm.

The transition from dense to dispersed pattern of integrin β6 implied the change of integrin activation after the initial adhesion formation. To determine the involvement of inside‐out or outside‐in signals, we undertook the domain‐swapping approach between integrin β6 and integrin β1, a well‐studied integrin receptor.^[^ ^29^ ^]^ Specifically, integrin β1 chimera with integrin β6's cytoplasmic tail (β1β6) and integrin β6 chimera with integrin β1's cytoplasmic tail (β6β1) were generated (**Figure** **2**A; Figure S2A, Supporting Information). When introduced to CHO‐B2 cells, both chimeras were located at the focal adhesion on RGD‐glass (RGD immobilized on glass; Figure S2B–E, Supporting Information). However, integrin β6β1, rather than integrin β1β6, formed persistent adhesions on viscous RGD‐membrane and colocalized with RGD clusters in 60‐min (Figure 2B). The decreasing slopes of linear regression in the pixel‐wise scatter plot also indicated a stronger association between RGD and integrin β6β1, compared to wildtype integrin β6 and β1β6 chimera (Figure 2C,D).

### 2.3 Kindlin2 Exhibits a Weak Association with Integrin β6

As integrin β6β1 enabled the persistent adhesion assembly, we sought to investigate the potential differences of adapter protein recruitment between integrin β6 and integrin β1. Quantitative PCR analysis indicated that talin1, kindlin1, and kindlin2 were expressed in CHO‐B2 cells, while talin2 and kindlin3 were nearly absent (Figure S2F, Supporting Information). Integrin cytoplasmic tails were synthesized and used as the bait to pull down adapter proteins from CHO‐B2 cell lysate. We found that similar levels of talin1 and kindlin1 were pulled down by integrin β6 and integrin β1 (Figure 2E–H). However, the levels of kindlin2 pulled down by integrin β6 were lower than those by integrin β1, while integrin β1^Y795A^ containing a point mutation in the membrane‐distal NxxY motif, as a negative control, failed to pull down kindlin2 (Figure 2I,J). Further supported by microscale thermophoresis (MST) measurement, the sigmoidal binding curves revealed that kindlin2 possessed a higher affinity for the cytoplasmic tail of integrin β1, compared to that of integrin β6, with a dissociation constant (K_d_) of 0.42 ± 0.11 and 26.60 ± 3.41 µM, respectively (Figure 2K,L; Figure S2G, Supporting Information).

### 2.4 Additional Kindlin2 Stabilizes Integrin β6 Clustering on Viscous RGD‐Membrane

**Figure 3:**
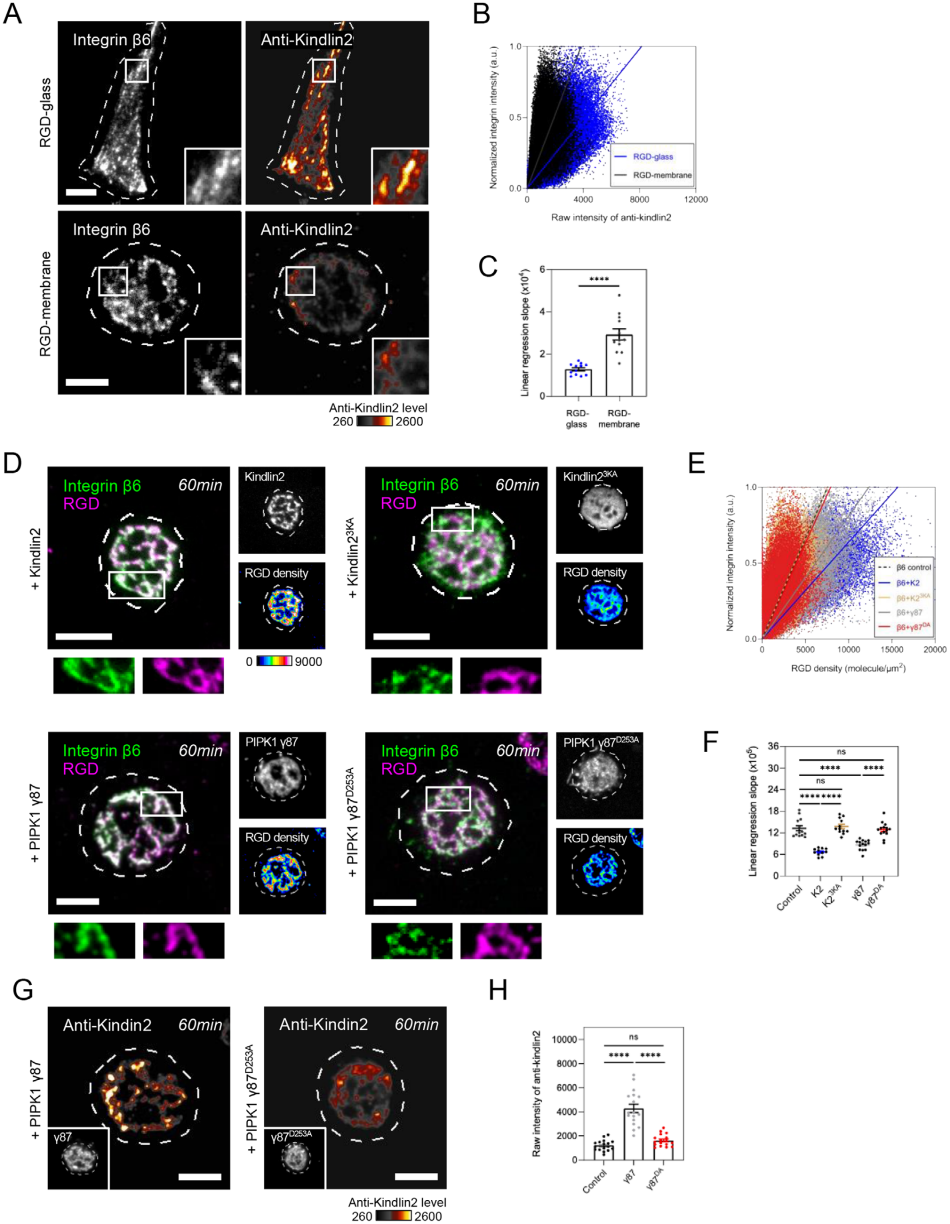
Kindlin2 reinforces the persistent assembly of RGD‐integrin β6 clusters. A) CHO‐B2 cells expressing integrin β6‐GFP form adhesions on RGD‐glass and RGD‐membrane. Pseudocolor panels indicate the level of endogenous kindlin2. The inset shows the boxed region (3.3 × 3.3 µM^2^). B) The scatter plot of anti‐kindlin2 and integrin intensity as indicated. C) The slopes of linear regression in B (cell number N; RGD‐glass, N=12; RGD‐membrane, N=12). D) Introductions of kindlin2 or PIPK1 γ87 stabilize integrin β6 adhesions. Neither kindin2^3KA^ (PH domain mutant) nor PIPK1 γ87^D253A^ (kinase‐dead mutant) promotes the persistency of integrin β6 adhesion. Pseudocolor panels indicate the corresponding densities of RGD ligand. The inset shows the boxed region (5 × 2 µM^2^). E) The scatter plot of RGD density and integrin intensity as indicated. The dashed line denotes the linear regression of integrin β6 control (see Figure 1D). F) The slopes of linear regression in E (cell number N; β6+K2, N=11; β6+K2^3KA^, N=12; β6+γ87, N=14; β6+γ87^D253A^, N=14). G,H) The levels of endogenous kindlin2 recruited to the adhesion in CHO‐B2 cells expressing PIPK1 γ87 or PIPK1 γ87^D253A^ (cell number N; β6, N=17; β6+γ87, N=17; β6+γ87^D253A^, N=17). All experiments have been independently repeated three times. Error estimates are S.E.M. The statistical information is in Table S4 (Supporting Information). Unpaired two‐tailed Student's *t*‐test and one‐way analysis of variance (ANOVA) are used for the statistical analysis. not significant, ns; *P* > 0.1234; and ^****^ *P* < 0.0001. Scale bars represent 5 µM.

We hypothesized that the weak association between integrin β6 and kindlin2 led to the dissociation of integrin β6 from RGD clusters. Indeed, the levels of endogenous kindlin2 recruited to integrin β6‐mediated adhesion on viscous RGD‐membrane were significantly lower than those on RGD‐glass (**Figure** **3**A). The slopes of linear regression in the pixel‐wise intensity scatter plot of anti‐kindlin2 and integrin β6 on RGD‐membrane also increased, in comparison with those on RGD‐glass (Figure 3B,C). Likewise, higher levels of endogenous kindlin2 were recruited to the RGD‐integrin β6 clusters during early adhesion at 15‐min than those of late adhesion at 60‐min (Figure S2H,I, Supporting Information). When additional wildtype kindlin2 was introduced, integrin β6 was able to form persistent adhesions on RGD‐membrane in 60‐min (Figure 3D–F). On the contrary, the expression of other adhesion adapters including talin1, paxillin, or vinculin had insignificant contributions to the stable adhesion formation of integrin β6 (Figure S2J–L, Supporting Information). It is known that the F3 domain of kindlin is the key motif to bind to the cytoplasmic tail of integrin β subunit. To further validate the specificity and rule out the overexpression artifact, we constructed two kindlin chimeras by exchanging the F3 domain between kindlin1 and kindlin2 (Figure S3A, Supporting Information) and confirmed their spatial enrichment at focal adhesions (Figure S3B–E, Supporting Information). When wildtype kindlin1 or the chimera of kindlin2 with kindin1's F3 domain (K2K1) was introduced, the dissociation of integrin β6 on viscous RGD‐membrane at 60‐min remained (Figure S3F–H, Supporting Information). On the other hand, the chimera of kindlin1 with kindin2's F3 domain (K1K2) effectively promoted the persistent adhesion assembly of integrin β6 (Figure S3F–H, Supporting Information). These data suggest that kindlin2 plays a critical role in supporting the adhesion assembly of integrin β6 on the traction force‐free substrate.

### 2.5 PIPK Elevates Kindlin2 Membrane Association and Strengthens RGD‐Integrin β6 Clustering

The interactions between kindlin and phosphoinositide in the plasma membrane are essential in kindlin‐mediated adhesion signaling.^[^ ^30^, ^31^ ^]^ When kindlin2^3KA^ (point mutations in the phosphoinositide‐binding pleckstrin homology (PH) domain, K385A, K386A, and K393A) was introduced, its membrane recruitment was defective, and integrin β6 was unable to form stable adhesions on the RGD‐membrane in 60‐min (Figure 3D; Figure S3I–N, Supporting Information). We further postulated that strengthening kindlin‐phosphoinositide interaction can promote the stability of integrin β6 adhesion. PIPK1 γ87 (also known as PIP5K γ635) is one of the key enzymes to promote the production of phosphatidylinositol‐4,5‐bisphosphate (PIP2) on the plasma membrane and is not specifically enriched at the adhesion site.^[^ ^32^ ^]^ When introducing PIPK1 γ87 rather than kinase‐dead mutant PIPK1 γ87^D253A^,^[^ ^33^ ^]^ integrin β6 was able to persistently nucleate RGD clusters in 60‐min (Figure 3D). The slopes of linear regression in the scatter plot of RGD density and integrin β6 also decreased in the presence of wildtype kindlin2 and PIPK1 γ87, compared to the control, kindlin2^3KA^, and PIPK1 γ87^D253A^ conditions (Figure 3E,F). Likewise, higher kindlin2 levels were evident at RGD adhesion clusters with additional PIPK1 γ87, in comparison with the control and PIPK1 γ87^D253A^ (Figure 3G,H). Thus, the reinforcement of kindlin‐phosphoinositide interaction effectively promotes the persistent adhesion of integrin β6.

### 2.6 Kindlin2 Promotes the Extended Conformation of Integrin β6 at RGD Clusters

**Figure 4:**
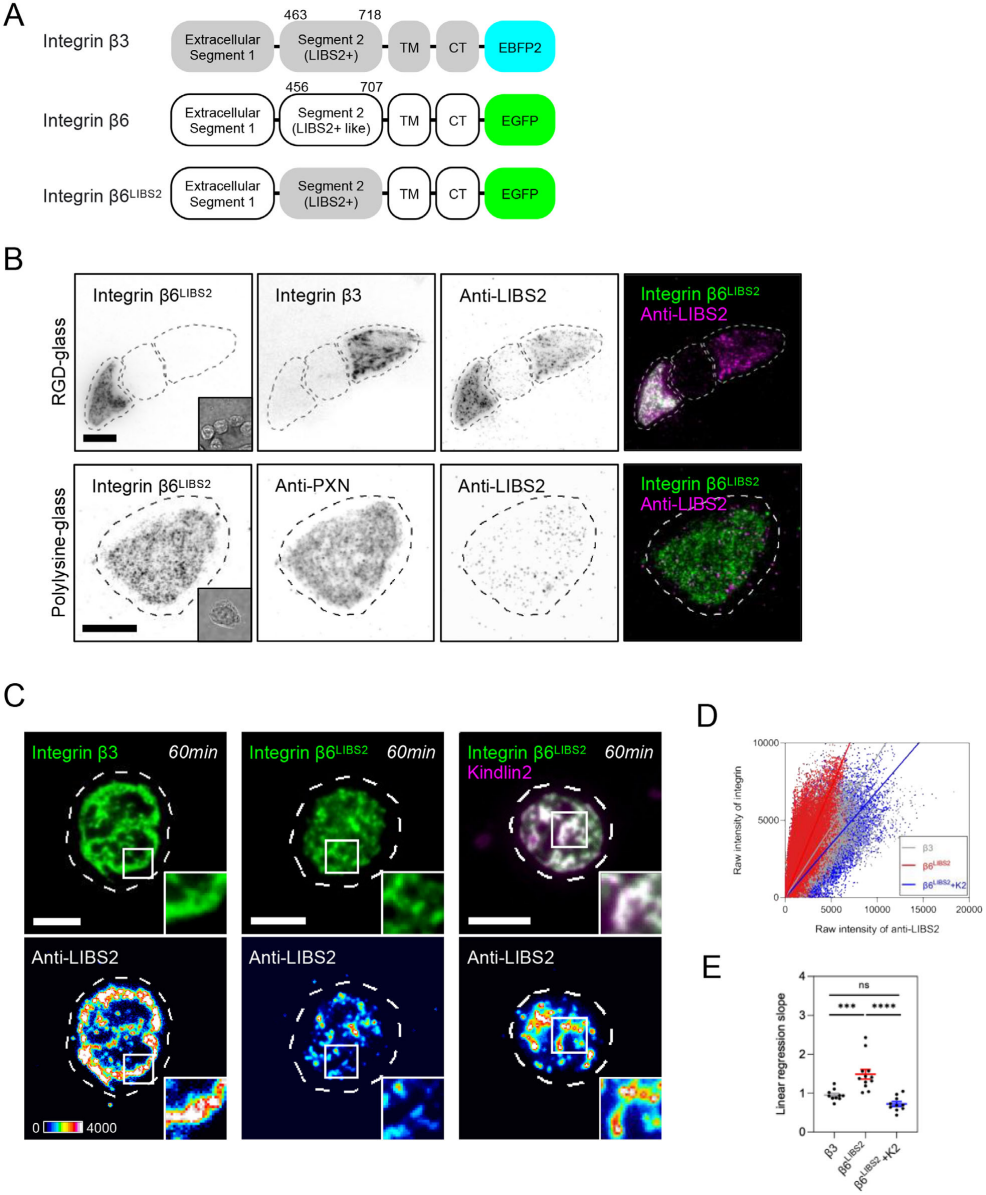
Kindlin2 promotes the active conformation of integrin β6. A) Schematic diagrams of integrin β3, β6, and LIBS2‐contaning chimera. B) Integrin β6^LIBS2^‐GFP and integrin β3‐EBFP2 are detected by anti‐LIBS2 antibody when CHO‐B2 cells adhere on RGD‐glass, rather than polylysine‐glass. C) CHO‐B2 cells with various integrin constructs form adhesions on RGD‐membrane and are fixed in 60‐min. Pseudocolor panels indicate the raw intensities of anti‐LIBS2. The inset shows the boxed region (2.8 × 2.8 µM^2^). D) The scatter plot of raw intensities of anti‐LIBS2 and integrins as indicated. E) The slopes of linear regression in D (cell number N; β3, N=10; β6^LIBS2^, N=12; β6^LIBS2^+K2, N=10). All experiments have been independently repeated three times. Error estimates are S.E.M. The statistical information is in Table S4 (Supporting Information). One‐way analysis of variance (ANOVA) is used for the statistical analysis. not significant, ns; *P* > 0.1234; ^***^ *P* < 0.0002; and ^****^ *P* < 0.0001. Scale bars represent 10 µM in B and 5 µM in C.

Conformational changes of integrin receptors, from the bent to the extended state, act as the hallmark of integrin activation. While conformation‐specific antibodies of several integrin subunits have been previously developed, such antibodies of integrin β6 are not available. As integrin β6 and integrin β3 share a high degree of similarity in domain homology, we employed the epitope‐substitution approach and generated an integrin β6 chimera that incorporated a detectable conformation‐specific epitope of integrin β3.^[^ ^34^ ^]^ Specifically, the homologous region in integrin β6 was substituted for the extracellular segment that contained the LIBS2 epitope of integrin β3, which is exposed in an extended conformation (**Figure** **4**A). When expressed in CHO‐B2 cells, LIBS2‐containing chimera integrin β6^LIBS2^ supported the adhesion formation on RGD‐glass and colocalized with focal adhesion markers (Figure S4A,B, Supporting Information). The conformation‐specific antibody anti‐LIBS2 was able to detect integrin β6^LIBS2^ in a similar fashion to integrin β3 (Figure 4B). Moreover, anti‐LIBS2 did not detect integrin β6^LIBS2^ when the cells were plated on polylysine‐coated glass (Figure 4B). It appears that anti‐LIBS2 can be used to detect the extended conformation of integrin β6^LIBS2^.

Next, we examined and confirmed that spatial reorganization of integrin β6^LIBS2^ and wildtype integrin β6 shared similar characteristics when cells adhered to RGD‐membrane, including dense clustering in 15‐min but dispersed pattern in 60‐min (Figure S4C–E, Supporting Information). In addition, anti‐LIBS2 weakly detected integrin β6^LIBS2^ in 60‐min, compared to wildtype integrin β3 (Figure 4C–E). When additional kindlin2 was expressed, more integrin β6^LIBS2^ were recognized by anti‐LIBS2. The slopes of linear regression in the pixel‐wise intensity scatter plot of anti‐LIBS2 and integrin β6^LIBS2^ also decreased when additional kindlin2 was introduced (Figure 4D,E). Thus, integrin β6 with the dispersed pattern on viscous RGD‐membrane is largely in the inactive conformation, and kindlin2 plays a critical role in promoting the activation and spatial clustering of integrin β6.

### 2.7 Kindlin2 Represses the Substrate Rigidity Dependence and Promotes Integrin β6‐Mediated Cell Migration

**Figure 5:**
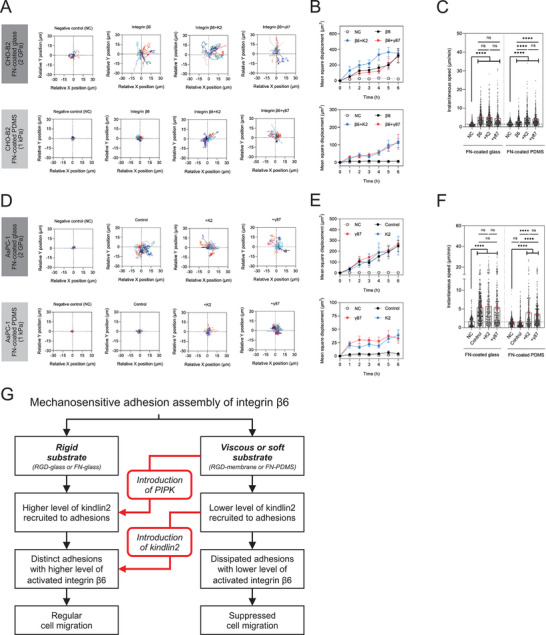
Kindlin2 bypasses the substrate rigidity dependence and promotes integrin β6‐mediated cell migration. A) Migration trajectories of CHO‐B2 cell for six hours. More than 10 cells are tracked under each indicated condition. B) Mean square displacement of cell migration in A. C) Instantaneous speed of cell migration in A. D) Migration trajectories of AsPC‐1 cell for six hours. More than 10 cells are tracked under each indicated condition. E) Mean square displacement of cell migration in D. F) Instantaneous speed of cell migration in D. G) Schematic summary. All experiments have been independently repeated three times. Error estimates are S.E.M. The statistical information is in Table S4 (Supporting Information). One‐way analysis of variance (ANOVA) is used for the statistical analysis. not significant, ns; *P* > 0.1234; and ^****^ *P* < 0.0001.

As the conformation of integrin β6 largely stayed in the inactive state on the viscous RGD‐membrane, we sought to examine the rigidity‐dependence of integrin β6‐mediated cell migration. Specifically, fibronectin‐coated glass (FN‐glass, 2 GPa) and soft polydimethylsiloxane (FN‐PDMS, 1 kPa) substrates were utilized.^[^ ^35^ ^]^ To avoid the contributions from other integrins expressed in CHO‐B2 cells, neutralizing antibodies against integrin αVβ5 (P1F6) were constantly applied. On FN‐glass, the migration of integrin β6‐expressed CHO‐B2 cells (CHO+β6) was suppressed when the neutralizing antibody of integrin αVβ6 (10D5; negative control) was applied. When 10D5 was absent, CHO+β6, as well as CHO+β6 with additional kindlin2 or PIPK1 γ87 had similar migration patterns in six hours and exhibited an instantaneous migration speed ≈5 µM min^−1^ (**Figure** **5**A–C). On the other hand, CHO+β6 cells on FN‐PDMS exhibited restricted migration patterns and showed similar mean square displacement to the condition of negative control. The addition of kindlin2 or PIPK1 γ87 to CHO+β6 cells then restored the migration capability on FN‐PDMS.

Integrin β6 has been identified as a molecular marker for various cancer cells, such as AsPC‐1 pancreatic adenocarcinoma cell (Figure S1A, Supporting Information). We sought to further validate the mechanism of integrin β6 mediated mechanosensitive migration in AsPC‐1 cells. Apart from integrin αVβ6, integrin αVβ5 and α5β1 were also expressed in AsPC‐1 cells. Thus, both neutralizing antibodies of integrin αVβ5 (P1F6) and integrin β1 (P4C10) were constantly applied, to investigate the specific role of integrin β6. On either FN‐glass or FN‐PDMS, the migration of AsPC‐1 cells was suppressed when the neutralizing antibody of integrin αVβ6 (10D5; negative control) was applied (Figure 5D–F). When 10D5 was absent, the migration of wildtype AsPC‐1 (control), as well as AsPC‐1 with additional kindlin2 or PIPK1 γ87 on FN‐glass recovered in an indistinguishable fashion. However, integrin β6 in the wild‐type AsPC‐1 cells did not support cell migration on FN‐PDMS, similar to CHO+β6 cells. Likewise, the addition of kindlin2 or PIPK1 γ87 to AsPC‐1 cells then restored the migration capability on FN‐PDMS. Taken together, these data suggest that kindlin2‐mediated activation of integrin β6 bypasses the substrate rigidity dependence and positively supports the cell migration on the compliant substrate.

## 3 Discussion

Integrin‐mediated adhesions are physical attachments between cells and extracellular space. Activations of integrin receptors, from the bent/inactive to extended/active conformation, are precisely regulated and require multiple steps, including extracellular ligand binding (outside‐in signals) and intracellular adapter protein association (inside‐out signals). Here, our results highlight the importance of both outside‐in and inside‐out signals. Nucleating extracellular ligand RGD is one of the initial events during the clustering of integrin β6 adhesion (15‐min) and indicates the pivotal role of outside‐in activation. In addition, we find that kindlin2 acts as the key cytoplasmic adapter protein to support the persistent clustering of integrin β6 on the traction force‐free RGD‐membrane over 60‐min. Other adapter proteins that can promote force‐dependent adhesion assembly, such as talin1, paxillin, and vinculin play a minor role under the traction force‐free condition. Our findings not only confirm the outside‐in activation of integrin β6 during the initial adhesion formation but also underline the important role of kindlin2 in maintaining the inside‐out activation and bypassing the mechanosensitive adhesion assembly of integrin β6 on the compliant substrate (Figure 5G).

The expression profile of various kindlins and their interaction with integrins are known to be tissue‐specific. Previous work based on mouse keratinocyte has reported the specific interaction between kindlin1 and integrin β6.^[^ ^36^ ^]^ Likewise, the work based on CHO‐A5 cells has revealed the differential association of kindlin2 with integrin β6 and integrin β1.^[^ ^37^ ^]^ In agreement with the literature, our in vitro pulldown and MST data demonstrate that kindlin2, rather than talin1 and kindlin1, exhibits a weak association with the cytoplasmic tail of integrin β6, compared to integrin β1. Intriguingly, endogenous kindlin2 can support persistent adhesion assembly of integrin β6 on rigid RGD‐glass but is insufficient to do so on viscous RGD‐membrane. As kindlin2 lacks direct interactions with the F‐actin cytoskeleton, this discrepancy of persistent adhesion assembly manifests the mechanosensitive characteristics of integrin β6 and suggests additional cytoskeletal‐independent pathways of kindlin2 recruitment, such as phosphoinositide biogenesis and adapter oligomerization.^[^ ^32^, ^38^, ^39^ ^]^

Previously, PH domain deleted mutant of kindlin2 has been used to demonstrate its interaction with phosphoinositides in the plasma membrane and is found to be weakly localized to the focal adhesion.^[^ ^31^, ^40^ ^]^ However, kindlin2's PH domain is embedded within the F2 domain, and the deletion of PH domain may disrupt the overall conformation and oligomerization of kindlin2.^[^ ^39^ ^]^ To avoid the deletion, we take the point‐mutation approach to generate the membrane‐binding deficient mutant kindlin2^3KA^ and demonstrate the critical role of kindlin2's membrane binding in promoting the persistent adhesion assembly of integrin β6 on RGD‐membrane. Likewise, elevated productions of PIP2 by additional PIPK1 γ87 enhance the recruitment of kindlin2 to RGD clusters and restore the persistent adhesion of integrin β6. PIP2 is an important lipid messenger at integrin‐mediated adhesions and acts as the precursor of phosphatidylinositol (3,4,5)‐trisphosphate [PI(3,4,5)P3], another key lipid messenger in cell migration and proliferation. It is plausible that the biogenesis of phosphoinositide is regulated in a mechanosensitive manner and orchestrates the overall level of kindlin2 recruitment to the plasma membrane.

Recent structure‐based works have demonstrated the homo‐dimerization of truncated kindlin2^[^ ^41^ ^]^ and homo‐trimerization of full‐length kindlin3.^[^ ^42^ ^]^ In general, the oligomerization of kindlin in the cytoplasm has been shown to play a critical role in the phase separation of adhesion complexes.^[^ ^39^ ^]^ The introduction of additional kindlin2 can compensate for its weak affinity to integrin β6 and potentially alters the homeostasis of phase‐separated adhesion complexes. Nevertheless, our data of kindlin1‐kindlin2 domain swapping underscore the importance of kindlin2's F3 domain, since K1K2 chimera, rather than wildtype kindlin1 or K2K1 chimera can reinforce the persistent assembly of RGD‐integrin β6 clusters. Further studies will be needed to unravel the functional role of kindlin oligomerization in mechanosensitive adhesion assembly.

Other signaling factors beyond kindlin2 may also be involved in the dissociation of integrin β6 on the traction force‐free viscous RGD‐membrane. Various kinases and phosphatases have been shown to regulate the assembly of traction force‐positive adhesion. For example, the tyrosine phosphorylation activities mediated by focal adhesion kinase (FAK) can positively stimulate the dynamic turnover of focal adhesion,^[^ ^43^ ^]^ while the tyrosine dephosphorylation activities of SHP2 promote the stability of focal adhesion.^[^ ^44^ ^]^ Likewise, tyrosine dephosphorylation activities of PTP1B and RPTPα can trigger Src activation, which in turn promotes FAK‐mediated adhesion remodeling and disassembly.^[^ ^45^ ^]^ Additional investigations are required to uncover how other adhesion signaling factors function when traction force is absent or in a compliant microenvironment.

Integrin inhibition has been one of the key approaches to block cancer metastasis, and higher expressions of integrin β6 are found in pancreatic, lung, bladder, and esophageal carcinomas.^[^ ^46^ ^]^ Our findings suggest that integrin β6 mostly stays in the inactive conformation when cells adhere to viscous RGD‐membrane. Likewise, the soft extracellular matrix can impair the integrin β6‐mediated migration in CHO+β6 cells and AsPC‐1 pancreatic cancer cells. While the adhesion assembly by integrin β6 is mechanosensitive, the introduction of kindlin2 can functionally bypass the mechanosensitivity and promote integrin β6‐mediated cell migration irrespective of the substrate rigidity. In general, kindlin‐mediated integrin activation demonstrated here highlights the crosstalk of inside‐out and outside‐in signals and can offer new insights into other mechanosensitive events related to cell adhesion and migration.

## 4 Experimental Section

### RGD‐Membrane

Detailed procedures were described previously.^[^ ^27^ ^]^ Lipid solution containing 1,2‐dioleoyl‐sn‐glycero‐3‐phosphocholine (DOPC, Avanti Polar Lipids 850375C) and 1,2‐dioleoyl‐sn‐glycero‐3‐phosphoethanolamine‐*N*‐(cap biotinyl) (Biotinyl‐Cap‐PE, Avanti Polar Lipids 870273C) were first dried in chloroform via rotatory evaporation under 60 °C, rehydrated with Milli‐Q water, and then probe sonicated in an ice bath. After one‐hour ultracentrifugation at 100 000 g, the supernatant containing small unilamellar lipid vesicles was collected and stored at 4 °C. Hydrophilic coverslip glass substrates (25 mm in diameter, #1.5) were prepared by bath sonication, prior to immersing in freshly prepared 50% sulfuric acid overnight. Before use, the coverslips were rinsed with Milli‐Q water and dried using a nitrogen gas stream. Except for the titration experiment, 99.8 mol% of DOPC and 0.2 mol% of biotinyl‐Cap‐PE in 30 µL of phosphate‐buffered saline (PBS) solution were introduced to the coverslip for five minutes at room temperature. Excess lipid vesicles were washed away in the water bath, and the coverslip coated with supported lipid membrane was placed in an Attofluor cell chamber (Thermo Fisher Scientific A7816). Membrane defects were blocked by 1% bovine serum albumin (BSA, Hyclone SH30574.02) for 30‐min. Following PBS washes, 1.5 µg of neutravidin with Dylight 594 (Thermo Fisher Scientific 22842) or Dylight 680 (Thermo Fisher Scientific 22848) was introduced for 30‐min. Excess neutravidin was rinsed off, followed by another 30‐min incubation of 1.5 µg of cyclo [Arg‐Gly‐Asp‐D‐Phe‐Lys(Biotin‐PEG‐PEG)] (RGD, Biosynth PCI‐3697‐PI). Unbound RGD was again removed by PBS washes. Each chamber was further rinsed with culture medium and kept at 37 °C upon cell seeding.

### Matrix Coating and PDMS Substrate

UV sterilized glass bottom dishes (#1.5, Mattek P35G‐1.5‐14‐C) were coated with fibronectin (FN, Gibco PHE0023, 10 µg mL^−1^) overnight at 4 °C or with poly‐D‐lysine (Gibco A3890401, 0.1 mg mL^−1^) for an hour at room temperature. For RGD‐functionalized glass substrate (RGD‐glass), clean coverslips were placed in the Attofluor chamber and incubated with 1 mg mL^−1^ of neutravidin overnight at 4 °C, followed by 1 mg mL^−1^ of biotinylated‐RGD for an hour at 37 °C. Before plating cells, coverslips were rinsed with PBS to remove any unbound proteins. Stiffness‐defined polydimethylsiloxane (PDMS, 1 kPa) substrates were prepared by spin‐coating the mixtures of Sylgard 527 on plasma‐cleaned coverslips at 500 rpm and were postbaked for 2 h at 80 °C.^[^ ^35^ ^]^ To perform cell migration assay, the PDMS substrates were coated with fibronectin as described above.

### Cell Culture

CHO‐B2,^[^ ^47^ ^]^ U2OS, and RPTPα^+/+^ mouse embryonic fibroblast (MEF; a gift from Dr. Jan Sap, NYU) cell lines were maintained using Dulbecco's Modified Eagle Medium (DMEM, Sigma D1152). AsPC‐1 cell line (a gift from Dr. Bo Gao, CUHK) was cultured using Roswell Park Memorial Institute (RPMI) 1640 medium (Sigma R6504). All cell lines were supplemented with 10% (v/v) fetal bovine serum (FBS, Hyclone SV30160.03) and 100 µg mL^−1^ penicillin‐streptomycin (Thermo Fisher Scientific 15140122) and cultured in a 37 °C incubator supplied with 5% CO_2_. Cells seeded on the RGD‐membrane were treated with 20 µM of ROCK inhibitor (Y‐27632, Selleckchem S1049) to decouple the intracellular contractility. Serum‐free L15 medium (Thermo Fisher Scientific 21083027) was used during live‐cell imaging. Unless otherwise mentioned, CHO‐B2 cells were used for investigation.

### Fluorescence Microscopy

Images were captured using an inverted total internal reflection fluorescence (TIRF) microscope or an inverted spinning disk confocal microscope. TIRF microscope (Nikon Eclipse Ti2‐E with iLAS3 TIRF module) equipped with a 100x oil immersion lens (NA = 1.49), Photometrics Prime95B camera, and acousto‐optic tunable filter (AOTF)‐modulated solid‐state lasers (405/488/561/640 nm, 50–100 mW) was controlled by MetaMorph (Molecular Devices). Spinning disk confocal microscope (UltraVIEW VoX; Nikon Eclipse Ti‐E with Yokogawa CSU‐X1) equipped with a 100x oil immersion lens (NA = 1.45), Hamamatsu C9100‐23B EMCCD camera, four AOTF‐controlled solid‐state lasers of 405/488/561/640 nm (40–50 mW) was controlled by Volocity (Perkin Elmer). Each microscope contained an on‐stage incubating chamber to maintain 37 °C and 5% CO_2_. Before imaging, all laser powers were calibrated by measuring the intensities of TetraSpeck microspheres immobilized on a coverslip. Images were taken in the 16‐bit format, and identical microscope parameters (equivalent laser power, camera gain, exposure time, and TIRF angle) were used to acquire the images for subsequent quantitative intensity analysis. To minimize pixel saturation and phototoxicity, low laser power and exposure time were applied during acquisition.

### RGD Density Titration and Quantification

Each neutravidin was assumed to be associated with two biotinyl‐Cap‐PE lipids on the supported bilayer membrane. The footprint area of each lipid molecule was previously reported and set as 0.72 nm^2^.^[^ ^48^ ^]^ When 99.8 mol% DOPC and 0.2 mol% biotinyl‐Cap‐PE lipids were used to form the supported bilayer membrane, the number of neutravidin per µM^2^ was estimated as 1389. After titrating the amount of biotinyl‐Cap‐PE lipids, images of fluorescently labeled neutravidin on the supported bilayer membrane were obtained by TIRF microscope using identical acquisition parameters. The intensities of fluorescently labeled neutravidin were measured in ImageJ. The linear regression between intensity and ligand density was generated by GraphPad Prism and used to convert the fluorescence intensity to the density of neutravidin‐RGD. While multiple biotinylated RGD ligands could bind to one neutravidin, the physical dimension of the ligand‐binding interface of the integrin receptor was larger than neutravidin.^[^ ^5^, ^49^ ^]^ Thus, only one of the RGD ligands on neutravidin was accessible to the integrin receptor.

### Reverse Transcription‐Quantitative Polymerase Chain Reaction (RT‐qPCR)

Total RNA was extracted with RNeasy Plus Mini Kit (Qiagen 74134) according to the manufacturer's instructions. RNA purity was measured with a NanoDrop2000c spectrophotometer (Thermo Fisher Scientific). PrimeScript RT reagent kit (Takara RR047A) was then used to yield cDNA templates. ChamQ SYBR color qPCR master mix (Vazyme Q411) was added to perform RT‐qPCR on a real‐time PCR detection system (Bio‐Rad, CFX96 Touch Deep Well). The relative gene expression was evaluated by using 2^‐ΔΔCt^ analysis method. The sequences of qPCR primers are listed in Table S1 (Supporting Information).

### DNA Constructs

Plasmids included integrin β6‐GFP (Addgene 13593), β5‐GFP (Addgene 205090), β3‐GFP,^[^ ^50^ ^]^ β1‐GFP (Addgene 69804), FERMT1_pLX307 (kindlin1, Addgene 98333), pEGFP‐kindlin2 (Addgene 105305), GFP‐PIPK1 γ87 (Addgene 22300), Lyn11‐FRB‐mCherry (Addgene 38004), GFP‐talin1 (Addgene 26724), pmCherry‐paxillin (Addgene 50526), pEGFP‐vinculin (Addgene 50513), EBFP2‐C1 (Addgene 54665) and pmiRFP703‐N1 (Addgene 79988). Integrin β1β6‐GFP, β6β1‐GFP, β6^LIBS2^‐GFP, BFP2‐K1K2, and BFP2‐K2K1 were constructed via Gibson assembly (Vazyme C115). Site‐directed mutagenesis was performed to generate BFP2‐kindlin2^3KA^ (K385A, K386A, K393A) and BFP2‐PIPK1 γ87^D253A^. The respective primer sequences can be found in Table S2 (Supporting Information). All plasmids were transiently transfected using Polyjet (SignaGen SL100688) and cells were used 48 h post transfection.

### Antibodies

Primary antibodies used for immunofluorescence (IF) and western blot (WB) were as follows: anti‐integrin β5 (R&D Systems AF3824; 1:200 for IF), anti‐paxillin (BD Biosciences 610569; 1:100 for IF), anti‐integrin β3, LIBS2 epitope clone ab62 (Merck MABT27; 1:50 for IF), anti‐kindlin2 (Proteintech 11453‐1‐AP; 1:100 for IF, 1:1000 for WB), anti‐kindlin2 clone 3A3 (Merck Millipore MAB2617; 1:1000 for WB), anti‐kindlin1 (Proteintech 22215‐1‐AP; 1:500 for WB), anti‐talin1 clone 8d4 (Merck T3287; 1:1000 for WB), and anti‐GAPDH (Thermo Fisher Scientific AM4300; 1:10000 for WB). Secondary antibodies included anti‐sheep‐AF594 (Thermo Fisher Scientific A‐11016; 1:800 for IF), anti‐mouse‐AF594 (Thermo Fisher Scientific A‐21203; 1:800 for IF), anti‐rabbit‐CF680R (Biotium 20195; 1:800 for IF), anti‐mouse‐HRP (Santa Cruz sc‐516102; 1:2000 for WB), and anti‐rabbit‐HRP (CST 7074; 1:2000 for WB). Anti‐integrin β5 clone KN52, PE (Thermo Fisher Scientific 12‐0497‐41; 1:20) was applied for flow cytometry. Anti‐integrin β1 clone P4C10 (Merck Millipore MAB1987; 1:100), anti‐integrin αVβ5 clone P1F6 (Merck Millipore MAB1961; 1:100), and anti‐integrin αVβ6 clone 10D5 (Merck Millipore MAB2077Z; 1:100) were used as the neutralizing antibodies in the cell migration experiment.

### Immunofluorescence Staining

Cells were fixed using 4% paraformaldehyde for 30‐min at 37 °C, and further permeabilized with 0.1% Triton X‐100 for another 30‐min at 37 °C. Next, 5% BSA in PBS was added for overnight blocking at 4 °C. Samples were subsequently incubated with primary antibody for 48 h at 4 °C, followed by secondary antibody incubation for two hours at room temperature after several PBS washes. Finally, samples were rinsed with PBS to remove any unbound antibodies.

### Pixel‐Wise Intensity Analysis

The camera background of the multi‐channel images was measured and then subtracted using ImageJ. The region of interest (ROI) of the entire cell was defined using the wand tool. Multi‐channel intensity values in every pixel within the ROI were measured using a customized ImageJ macro. In general, around few thousand data points were collected from the pixel‐wise analysis of a single cell. No image binarization process was utilized, to retain the dynamic range of intensity level. The scatter plots and linear regressions of integrin intensity were generated by GraphPad Prism. The linear regression of integrin β6 control (Figure 1D, 60‐min) was used as a reference to compare the spatial clustering in indicated conditions.

### Synthetic Peptide and Pulldown

The cytoplasmic tail peptides, including wildtype integrin β6 and β1, as well as integrin mutant β1^Y795A^ were purchased from GL Biochem (Shanghai, China) and *N*‐terminally biotinylated. Pulldown experiments were based on CHO‐B2 cell lysates and conducted in accordance with a standard protocol provided by the manufacturer (Pierce Biotinylated Protein Interaction Pull‐Down Kit, Thermo Fisher Scientific 21115). The peptide sequences can be found in Table S3 (Supporting Information).

### Kindlin2 Expression and Purification

Full‐length mouse kindlin2 with 10xHis‐SUMO tag and HRV‐3C site at the N‐terminal was cloned into pET28a vector and expressed in *Escherichia coli* Rosetta(DE3) cells. Kindlin2 expression was induced by the addition of 500 mm IPTG overnight at 18 °C. The protein was purified using immobilized metal chelate affinity chromatography (IMAC) in a high‐salt TBS buffer (20 mm Tris, pH 7.5, 500 mm NaCl, 1 mm Tris(2‐carboxyethyl)phosphine (TCEP)). The 10xHis‐SUMO tag was then cleaved by HRV 3C Protease (Takara 7360) overnight. The cleaved product was cleaned via size‐exclusion chromatography (SEC) with TBS (20 mm Tris, pH 7.5, 200 mm NaCl, 1 mm TCEP) containing 5% glycerol as both the running and storage buffer.

### Microscale Thermophoresis Assay

MST measurements were conducted using a Monolith NT.115 red‐blue machine (NanoTemper) with capillaries (NanoTemper MO‐K022‐SP). FITC‐labeled cytoplasmic tails of wildtype integrin β6, β1 and β1^Y795A^ were synthesized by Sangon Biotech. The integrin β tail peptides and kindlin2 protein were resolved in the MST buffer (20 mm Tris, pH 7.5, 200 mm sodium chloride, 1 mM TCEP, 5% glycerol) and measurements were performed at 10–20% LED power. The concentrations of the tail peptides were adjusted to 10 µM constantly and the concentration of kindlin2 ranged from 42.5 to 0.0013 µM. Data analysis was carried out via MO. Affinity Analysis 3 software using the K_d_ model for curve fitting.

### Western Blotting

Cell lysates were harvested in RIPA buffer (Thermo Fisher Scientific 89900) containing EDTA‐free protease and phosphatase inhibitors (Thermo Fisher Scientific A32965), and further boiled in 4x Laemmli buffer (Biorad 1610747) for 10‐min at 95 °C. Protein concentration was determined using Pierce BCA Protein Assay Kit (Thermo Fisher Scientific 23225). Next, protein samples were separated on sodium dodecyl sulfate‐polyacrylamide gel electrophoresis (SDS‐PAGE) gel, transferred to polyvinylidene difluoride (PVDF) membrane, and blocked with 5% non‐fat dry milk (Blotto, Santa Cruz Biotechnology sc‐2324) in Tris‐buffered saline with Tween 20 (TBST) for an hour at room temperature. PVDF membrane was then incubated with the primary antibody overnight at 4 °C. Afterward, PVDF membrane was rinsed with TBST and incubated with the corresponding secondary antibody for an hour at room temperature. Finally, SuperSignal West Femto Maximum Sensitivity Substrate (Thermo Fisher Scientific 34096) was applied to develop the blots.

### Flow Cytometry

Flow cytometry was conducted on the NovoCyte Advanteon BVYG flow cytometer (Agilent) following the standard procedures. CHO‐B2 cells were first collected, washed twice with ice‐cold PBS, and then incubated with PE‐labeled integrin β5 antibody in PBS containing 3% FBS, for 30‐min at 4 °C. The relative expression of cell surface integrin β5 was assessed using the FlowJo software.

### Membrane Association Assay

Cells co‐transfected with Lyn11 (a plasma membrane marker) and wildtype kindlin2, or kindlin^3KA^, were trypsinized and resuspended in L15 medium before imaging. Cells were then introduced over a non‐coated glass bottom dish. The images at the focal plane of cell great circle, marked by Lyn11, were captured by a spinning disk confocal microscope. Line‐scan across the great circle was then generated using ImageJ, and corresponding intensities were plotted with GraphPad Prism.

### Migration Assay

Before plating onto fibronectin‐coated glass or fibronectin‐coated PDMS substrate, CHO‐B2 cells were incubated with an anti‐αVβ5 neutralizing antibody, whereas AsPC‐1 cells were incubated with both anti‐αVβ5 and anti‐β1 neutralizing antibodies. An additional anti‐αVβ6 neutralizing antibody was included in the negative control. Cell permeable far‐red fluorescent DNA dye DRAQ5 (Thermo Fisher Scientific 62251) was used to locate the cell during the migration. Tiled images (5 × 5) were acquired every 10‐min using a 20x objective lens on a spinning disk confocal microscope. The x‐y trajectories, mean square displacement (MSD), and the instantaneous speed of migrating cells, indicated by the DRAQ5‐labeled nuclei, were analyzed using Imaris and then plotted in GraphPad Prism.

### Statistical Tests

Statistical graphs showing the mean and standard error of the mean (S.E.M.) were plotted using GraphPad Prism. At least three biological repeats were independently carried out for all experiments. Unpaired two‐tailed Student's t‐test and one‐way analysis of variance (ANOVA) were adapted for statistical analyses. Detailed statistical information including the P values are displayed in Table S4 (Supporting Information). not significant, ns; *P* > 0.1234; ^*^ *P* < 0.0332; ^**^ *P* < 0.0021; ^***^ *P* < 0.0002; and ^****^ *P* < 0.0001.

## Conflict of Interest

The authors declare no conflict of interest.

## Author Contributions

W.N.L. and C.H.Y. discussed the experiments and wrote the manuscript. C.H.Y. and N.P.C. supervised and provided a conceptual design of the research project. W.N.L. performed most of the experiments and data analyses. J.L. conducted the microscale thermophoresis experiment and processed the data. N.P.C. and C.H.Y. evaluated the experimental settings and data interpretation.

## Supporting information



Supporting Information

Supporting Table

## Data Availability

The data that support the findings of this study are available from the corresponding author upon reasonable request.
