# Supplementary material for: Kindlin Regulates Mechanosensitive Activation and Adhesion Assembly of Integrin beta6
Source: Adv Sci (Weinh). 2025 Jun 10;12(32):e01078. doi: 10.1002/advs.202501078 (PMC12407373; doi:10.1002/advs.202501078)
Supplement: Supplementary file 1 — Supporting Information [file ADVS-12-e01078-s002.pdf]

ADVANCED SCIENCE
Open Access

Supporting Information

for *Adv. Sci.*, DOI 10.1002/advs.202501078

Kindlin Regulates Mechanosensitive Activation and Adhesion Assembly of Integrin beta6

Wan Ning Lee, Jiamin Li, Nan-Peng Chen and Cheng-han Yu**

Supplemental figure

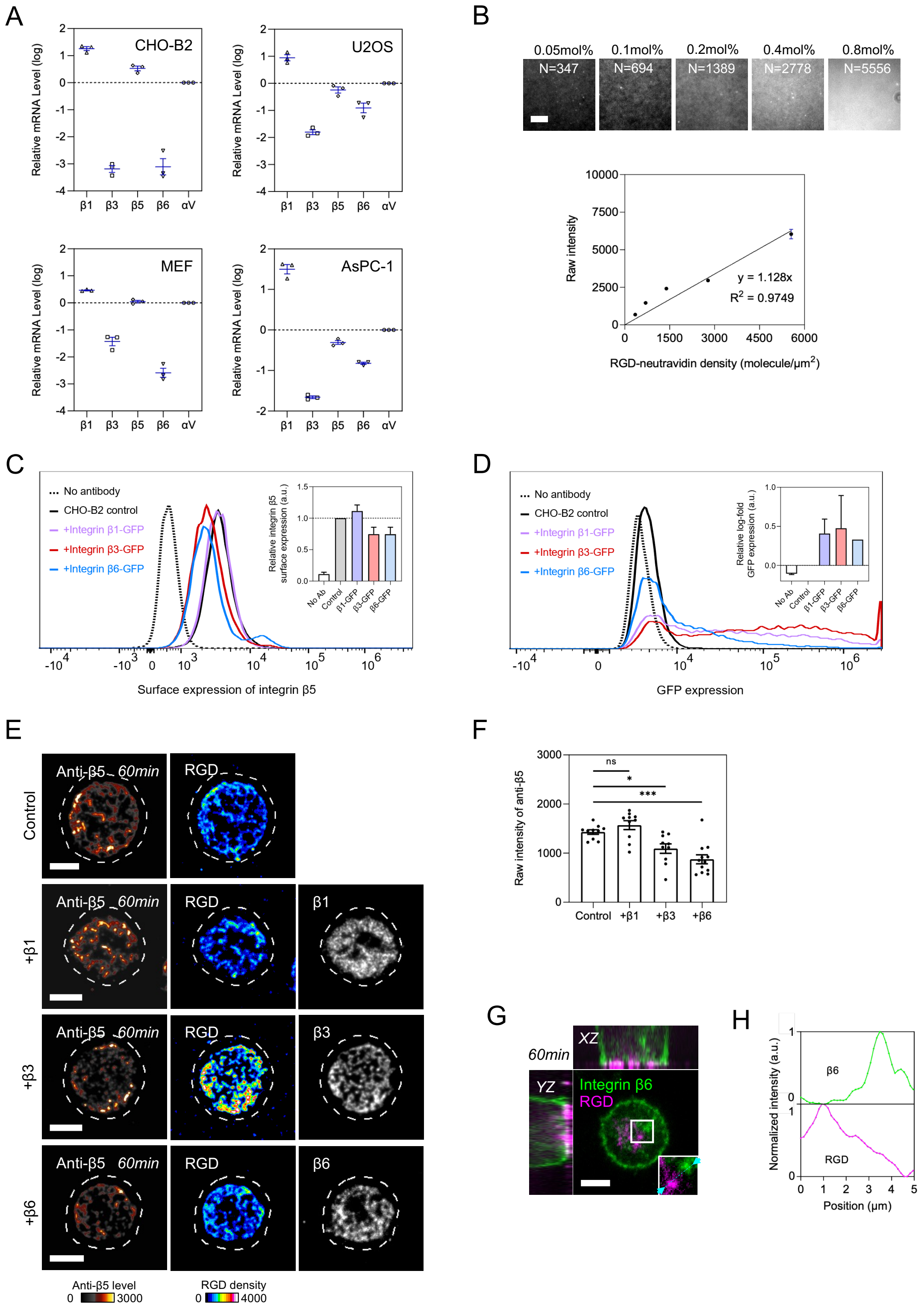

Figure S1. (A) The RT-qPCR analysis of integrin expression. Integrin β1, β5, and αV are expressed in CHO-B2, U2OS and MEF cells, whereas integrin β3 and β6 are at

non-detectable levels. AsPC-1 pancreatic adenocarcinoma cells natively express integrin β6. (B) Images and intensity-density calibration of Dylight 594 labeled neutravidin on the RGD-membrane of indicated biotinylated lipid density (See Methods). (C) Integrin β5 cell surface expression in CHO-B2 cells of each indicated condition determined by flow cytometry. Additions of integrin β3 or β6 can promote the dimerization with the endogenous integrin αV and lead to a small reduction of integrin β5 on the plasma membrane. Conversely, additional integrin β1 results in insignificant changes. (D) Expression levels of EGFP-tagged integrins in C. (E-F) The level of endogenous integrin β5 at the RGD clusters in 60 minutes. Pseudocolor panels indicate the raw intensities of anti-β5 (left) and the corresponding densities of RGD ligand (middle). (G) Confocal images of integrin β6 and endocytosed RGD ligands in 60 minutes. The inset shows the boxed region (3.3×3.3 μm^2). (H) Intensity line scan along the dashed line in G. Integrin β6 does not colocalize with the endocytosed RGD puncta. All experiments have been independently repeated three times. Error estimates are S.E.M. The statistical information is in Table S4. One-way analysis of variance (ANOVA) is used for the statistical analysis. not significant, ns; $P > 0.1234$; $^{*}P < 0.0332$; and $^{***}P < 0.0002$. Scale bars represent 5 μm.

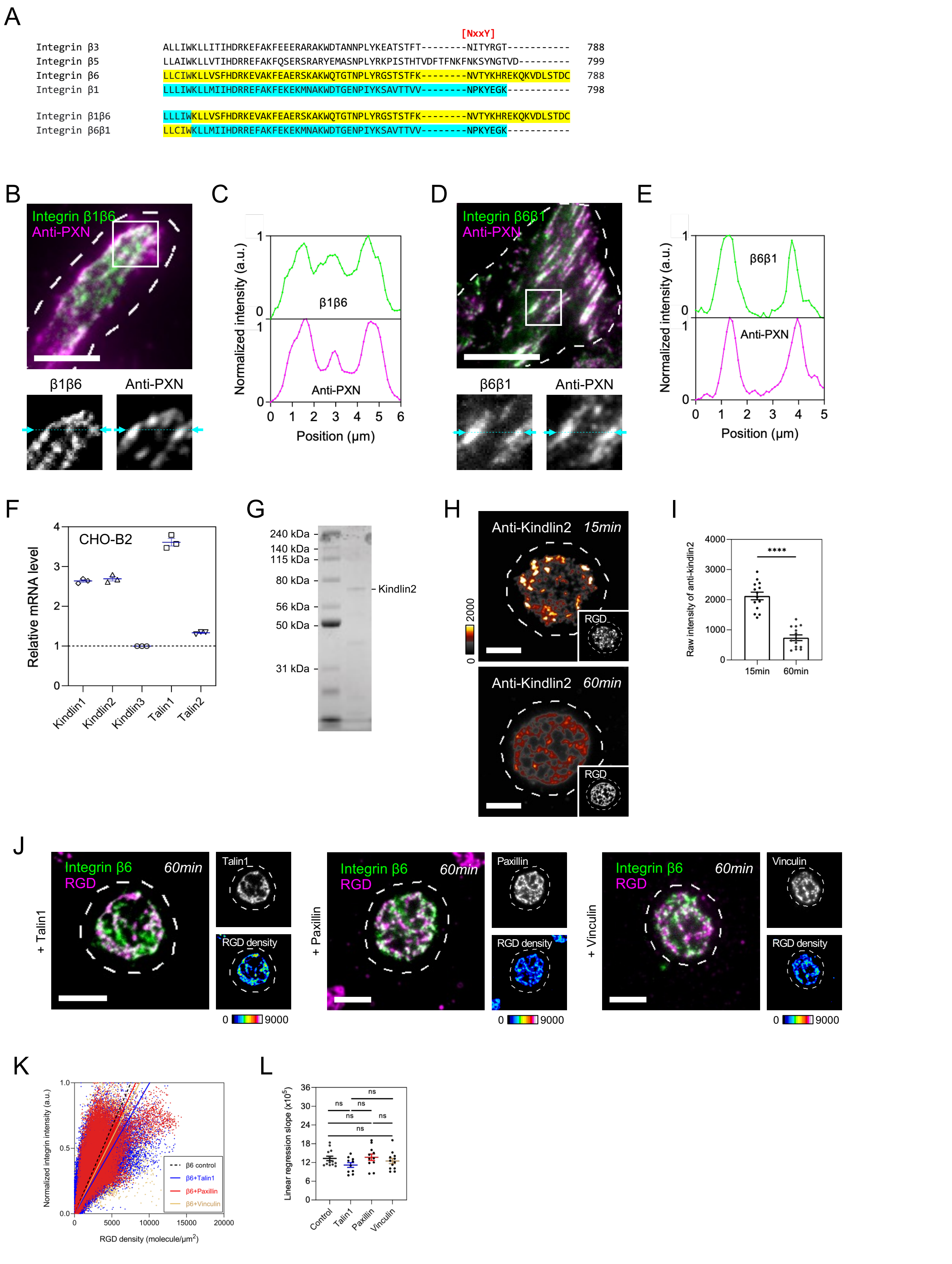

Figure S2. (A) Sequence alignment of integrin β3, β5, β6, β1, and domain swapping chimeras. (B) Integrin β1β6-GFP is located at the focal adhesion and colocalizes with paxillin when CHO-B2 cells adhere on immobile RGD-glass. The inset shows the

boxed region (4.4×4.4 μm^2). (C) Intensity line scan along the dashed line in B. (D) Integrin β6β1-GFP is located at the focal adhesion and colocalizes with paxillin when CHO-B2 cells adhere on immobile RGD-glass. The inset shows the boxed region (4.4×4.4 μm^2). (E) Intensity line scan along the dashed line in D. (F) The RT-qPCR analysis of various kindlin and talin isoforms. CHO-B2 cells mainly express kindlin1, kindlin2, and talin1. (G) Western blot of purified kindlin2 for the MST experiment. (H-I) The levels of endogenous kindlin2 recruited to the adhesion in CHO-B2 cells in 15 and 60 minutes (cell number N; 15-min, N=14; 60-min, N=14). (J) Additional GFP-talin1, mCherry-paxillin, or GFP-vinculin fails to restore the dense RGD-integrin β6 clustering in 60 minutes. Pseudocolor panels indicate the corresponding densities of RGD ligand. (K) The scatter plot of RGD density and integrin intensity as indicated. The dashed line denotes the linear regression of integrin β6 control (See Fig. 1D). (L) The slopes of linear regression in N (cell number N; β6+Talin1, N=10; β6+Paxillin, N=11; β6+Vinculin, N=12). All experiments have been independently repeated three times. Error estimates are S.E.M. The statistical information is in Table S4. Unpaired two-tailed Student's t-test and one-way analysis of variance (ANOVA) are used for the statistical analysis. not significant, ns; $P > 0.1234$; and ****$P < 0.0001$. Scale bars represent 10 μm in B, D and 5 μm in H and J.

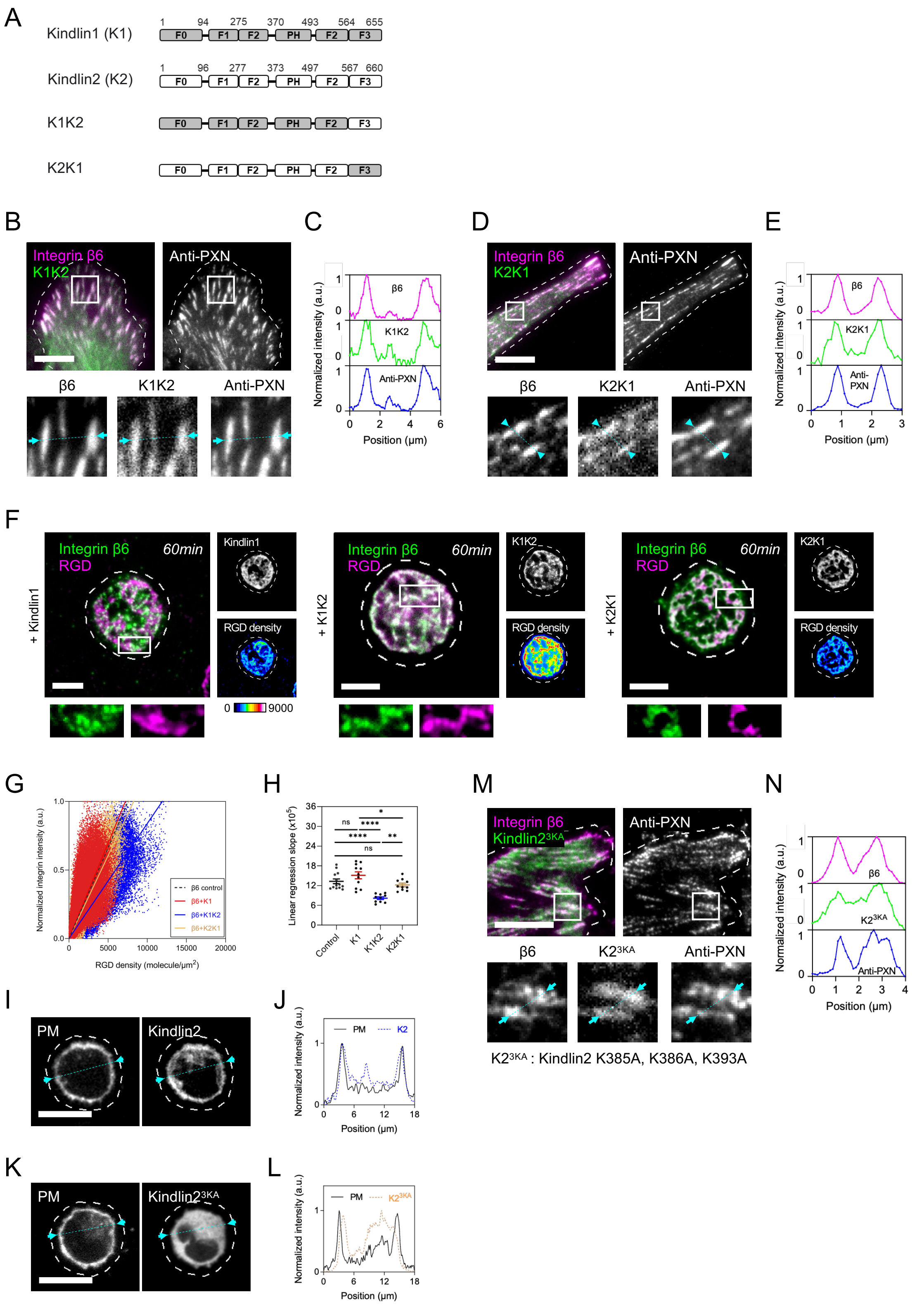

Figure S3. (A) Schematic diagrams of kindlin1, kindlin2 and domain swapping chimeras. (B) BFP2-K1K2 colocalizes with integrin β6-GFP at the focal adhesion when CHO-B2 cells adhere on RGD-glass. Paxillin is used to mark focal adhesions.

The inset shows the boxed region (6.6×6.6 μm^2). (C) Intensity line scan along the dashed line in B. (D) BFP2-K2K1 colocalizes with integrin β6-GFP at the focal adhesion when CHO-B2 cells adhere on RGD-glass. Paxillin is used to mark focal adhesions. The inset shows the boxed region (4.4×4.4 μm^2). (E) Intensity line scan along the dashed line in D. (F) Introduction of K1K2, rather than wildtype kindlin1 or K2K1, stabilizes integrin β6 adhesions. Pseudocolor panels indicate the corresponding densities of RGD ligand. The inset shows the boxed region (5×2 μm^2). (G) The scatter plot of RGD density and integrin intensity as indicated. The dashed line denotes the linear regression of integrin β6 control (See Fig. 1D). (H) The slopes of linear regression in N (cell number N; β6+K1, N=11; β6+K1K2, N=10; β6+K2K1, N=10). (I) Confocal images of BFP2-kindlin2 taken at the great circle plane of suspended CHO-B2 cell. Lyn11-iRFP is used to mark the plasma membrane (PM). (J) Intensity line scan along the dashed line in I. (K) Confocal images of BFP2-kindlin2^{3KA} taken at the great circle plane of suspended CHO-B2 cell. (L) Intensity line scan along the dashed line in K. Kindlin2^{3KA} is not associated with the plasma membrane. (M) Kindlin2^{3KA} is rather diffusive and weakly located at the focal adhesion when CHO-B2 cells adhere on RGD-glass. Paxillin is used to mark focal adhesions. The inset shows the boxed region (4.4×4.4 μm^2). (N) Intensity line scan along the dashed line in M. All experiments have been independently repeated three times. Error estimates are S.E.M. The statistical information is in Table S4. One-way analysis of variance (ANOVA) is used for the statistical analysis. not significant, ns; $P > 0.1234$; $^*P < 0.0332$; $^{**}P < 0.0021$; and $^{****}P < 0.0001$. Scale bars represent 10 μm in B, D, I, K, M and 5 μm in F.

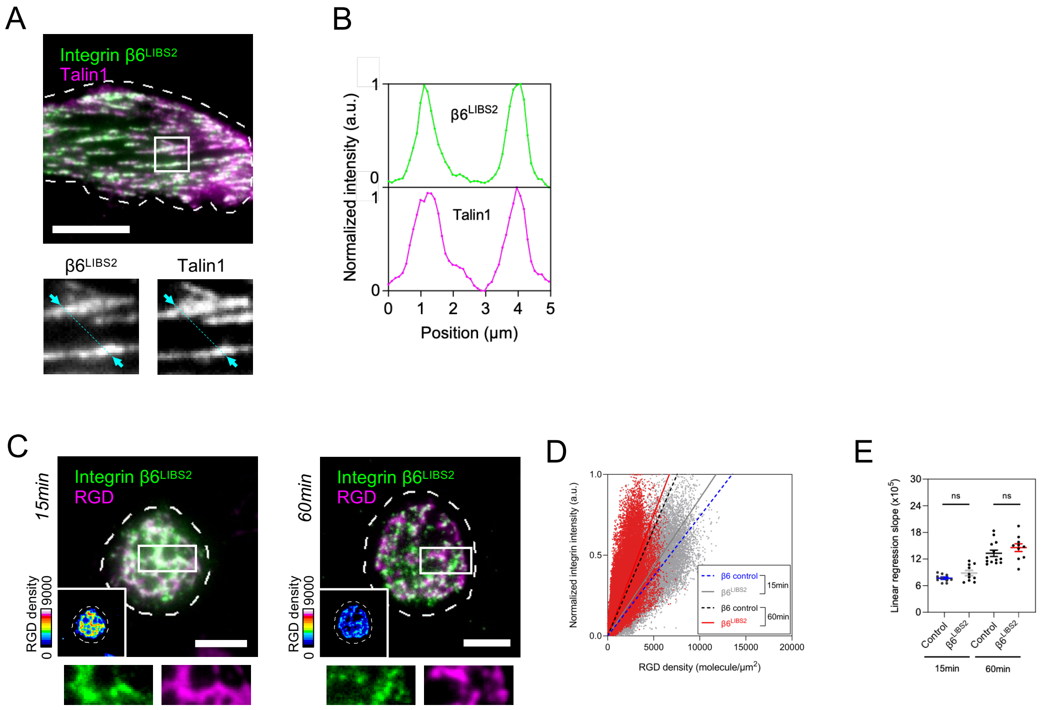

Figure S4. (A) Integrin β6^{LIBS2}-GFP is located at the focal adhesion marked by BFP2-talin1 when CHO-B2 cells adhere on RGD-glass. The inset shows the boxed region (4.4×4.4 μm^2). (B) Intensity line scan along the dashed line in A. (C) Integrin β6^{LIBS2}-GFP forms dense adhesions in 15 minutes and then dissociates from RGD clusters in 60 minutes. Pseudocolor panels indicate the corresponding densities of RGD ligand. The inset shows the boxed region (5×2 μm^2). (D) The scatter plot of RGD density and integrin intensity in 15 and 60 minutes. The dashed lines denote the linear regression of integrin β6 control (See Fig. 1B and 1D). (E) The slopes of linear regression in D (integrin β6^{LIBS2} cell number N; 15-min, N=10; 60-min, N=10). All experiments have been independently repeated three times. Error estimates are S.E.M. The statistical information is in Table S4. One-way analysis of variance (ANOVA) is used for the statistical analysis. not significant, ns; P > 0.1234. Scale bars represent 10 μm in A and 5 μm in C.

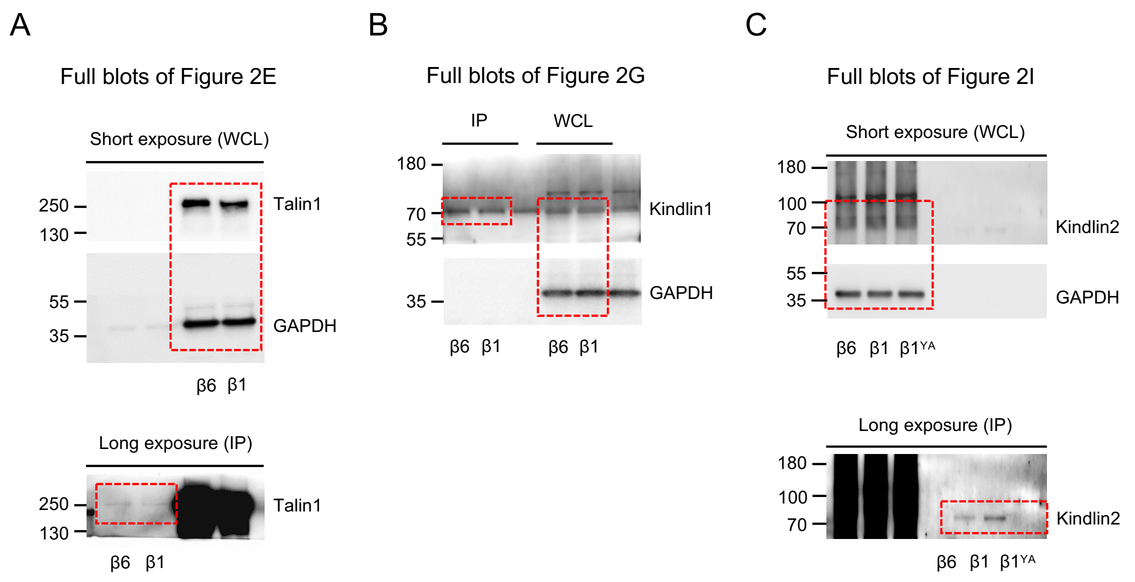

Figure S5. (A-C) Original western blots of integrin pulldown experiments. WCL, whole cell lysate; IP, immunoprecipitation.
